# Antimicrobial resistance and whole genome sequencing of novel sequence types of *Enterococcus faecalis, Enterococcus faecium,* and *Enterococcus durans* isolated from livestock

**DOI:** 10.1038/s41598-023-42838-z

**Published:** 2023-10-30

**Authors:** Mohamed E. El Zowalaty, Bibek Lamichhane, Linda Falgenhauer, Shakeel Mowlaboccus, Oliver T. Zishiri, Stephen Forsythe, Yosra A. Helmy

**Affiliations:** 1 Veterinary Medicine and Food Security Research Group, Medical Laboratory Sciences Program, Faculty of Health Sciences, Abu Dhabi Women’s Campus, Higher Colleges of Technology, Abu Dhabi, 41012 UAE; 2 Department of Veterinary Science, Martin-Gatton College of Agriculture, Food, and Environment, University of Kentucky, Lexington, KY 40546 USA; 3 Institute of Hygiene and Environmental Medicine, Justus Liebig University Giessen, Biomedical Research Center Seltersberg, Schubertstrasse 81, 35392 Giessen, Germany; 4 Antimicrobial Resistance and Infectious Diseases Research Laboratory, College of Science, Health, Engineering and Education, Murdoch University, Murdoch, WA Australia; 5 Department of Microbiology, PathWest Laboratory Medicine-WA, Fiona Stanley Hospital, Murdoch, WA Australia; 6 Discipline of Genetics, School of Life Sciences, College of Agriculture, Engineering and Science, University of KwaZulu-Natal, Private Bag X54001, Westville, Durban, 4000 South Africa; 7 Foodmicrobe.com Ltd., Adams Hill, Keyworth, Nottingham, NG12 5GY UK; 8 Department of Zoonoses, Faculty of Veterinary Medicine, Suez Canal University, Ismailia, 41522 Egypt

**Keywords:** Bacteriology, Infectious-disease epidemiology, Bacterial genomics

## Abstract

The emergence of antimicrobial-resistant, livestock-associated *Enterococcus faecalis* represents a public health concern. Here, we report the isolation, molecular detection of virulence and antimicrobial resistance determinants, in addition to the phylogenetic analyses of 20 *Enterococcus* species using whole genome sequencing analysis of 15 *Enterococcus faecalis* strains including six strains of three novel sequence types, three *Enterococcus faecium* and two *Enterococcus durans*. All strains were isolated from food chain animals in South Africa. *Enterococcus* strains were isolated on bile aesculin azide agar, followed by identification using MALDI-TOF MS analysis. Antibiotic susceptibility testing was performed using the Kirby–Bauer disk diffusion method. The genomic DNA of the isolates was extracted and sequencing was performed using the Illumina MiSeq platform. Sequence reads were trimmed and de novo assembled. The assembled contigs were analyzed for antimicrobial resistance genes and chromosomal mutations, extra-chromosomal plasmids, and multi-locus sequence type (MLST). Multidrug antimicrobial resistance genes conferring resistance to aminoglycosides (*ant(6)-Ia*, *aph(3′)-IIIa*, *sat4,* and *spw*), lincosamides (*lnu(B)*, *lsa(A)*, and *lsa(E)*), macrolides (*erm(B)*), trimethoprim (*dfrG*) and tetracyclines (*tet(L)* and *tet(M)*) were identified. Plasmid replicons were detected in seven *E. faecalis* and three *E. faecium* isolates. The sequence type (ST) of each isolate was determined using the *Enterococcus* PubMLST database. Ten STs were identified in the collection, three of which (ST1240, ST1241, and ST1242) have not been previously reported and are described in the present study for the first time. To compare the sequenced strains to other previously sequenced *E. faecalis* strains, assembled sequences of *E. faecalis* from livestock were downloaded from the PubMLST database. Core genome-based phylogenetic analysis was performed using ParSNP. The detection of multiple drug-resistance in *Enterococcus* including *E. faecalis* and *E. faecium* highlights the significance of genomic surveillance to monitor the spread of antimicrobial resistance in food chain animals. In addition, the genome sequences of *Enterococcus* strains reported in the present study will serve as a reference point for future molecular epidemiological studies of livestock-associated and antibiotic-resistant *E. faecalis* in Africa. In addition, this study enables the in-depth analysis of *E. faecalis* genomic structure, as well as provides valuable information on the phenotypic and genotypic antimicrobial resistance, and the pathogenesis of livestock-associated *E. faecalis* and *E. faecium*.

## Introduction

*Enterococcus* species are Gram-positive, non-spore forming cocci which naturaly occur in healthy humans and animals. *Enterococcus* remain one of the most important zoonotic enteric pathogens that are responsible for morbidity and mortality in humans and livestock worldwide. They also serve as indicator bacteria in the surveillance for antimicrobial resistance in food chain animals^1^. *Enterococcus* spp. are important commensal bacteria of the normal gut flora of humans and animals^2^, however, they have recently emerged as leading causes of zoonotic and nosocomial infections^3–6^. Vancomycin-resistant enterococci (VRE) are among the critical-priority resistant bacteria in the “global priority list of antibiotic-resistant bacteria for the research and development of new antibiotics”^1,7^. *Enterococcus faecium* and *Enterococcus faecalis* are the most common nosocomial opportunistic pathogens responsible for approximately 10–15% and 80–90% of infections, respectively^8^. *E. faecalis* is an important zoonotic enteric pathogen that may cause serious infections in humans and animals, including gastrointestinal tract infections and extra-intestinal infections including urinary tract infections, meningitis, bacteremia, peritonitis, endocarditis, and periodontitis^9–11^. *E. faecalis* infections primarily spread from person to person through poor hygiene. The bacteria can contaminate foods through inadequate hygiene and food manipulation. Foodstuffs, particularly ready-to-eat foods and foods of animal origins, may be the sources of bacterial transmission supporting their zoonotic potential^12–15^.

Studies investigating food animals as a zoonotic reservoir for common human bacterial pathogens have confirmed the zoonotic origin of human bacterial pathogens such as *Salmonella* spp.^16^, *Campylobacter* spp.^17^ and pathogenic *Escherichia coli*^18,19^. These studies also highlight the possible zoonotic origin for other major human bacterial infections involving *Staphylococcus aureus*, *E. faecalis*, and *E. faecium*^9^. Food animals meat production settings and livestock production systems are important sources of antimicrobial resistance genes selected by the use of antibiotics, carried from poultry and livestock to humans by zoonotic bacterial clones. These resistance factors can be transferred to other bacteria present in the gut microbiota of the consumer through horizontal gene transfer^1,7,9^.

*Enterococcus* is responsible for a significant proportion of multidrug-resistant intestinal and extra-intestinal infections in humans and food animals. Virulent and multidrug-resistant strains of *Enterococcus* have been detected and isolated from domesticated animals including bovine, poultry, pigs, and pets as well as food products from different regions^12^. The detection and worldwide spread of resistance genes including the colistin *mcr-1* genes in livestock and humans have spurred efforts of genomic surveillance for antimicrobial resistance in food animals^20^.

Genome sequence analysis of *Enterococcus* is essential because of the potential impact of these bacteria on human and animal health. Whole genome analysis is an ideal approach to detect genomic variations, and provides valuable information enabling us to understand virulence, pathogenesis, antimicrobial resistance, host specificity and phylogenetic relationships^21–23^. However, currently there is very little data concerning the genome sequence of *Enterococcus* spp. isolated from livestock in Africa are available. In addition, data on genetic characterization of *Enterococcus* spp from the livestock production sector in South Africa are limited, and whole genome sequencing is not routinely employed to evaluate virulence and AMR associated genes^24^. Here, we report the use of whole genome sequencing in analysis of fifteen *E. faecalis*, three *E. faecium,* and two *E. durans* strains isolated from different livestock animals (cattle, chicken, goat, horse and pigs) in KwaZulu-Natal and Eastern Cape Provinces in South Africa.

## Materials and methods

### Ethical statement

All procedures and methods were carried out in accordance with relevant guidelines and regulations and were reported in accordance with ARRIVE guidelines (https://arriveguidelines.org). No anesthesia or euthanasia methods were employed or involved in the present study. The study was approved by the Animal Research Ethics Committee of the University of Kwa-Zulu Natal (Reference numbers AREC/051/017 M, AREC 071/017 and AREC014/018). The field sampling protocols, samples collected from animals, and the research were conducted in full compliance with Section 20 of the Animal Diseases Act of 1984 (Act No 35 of 1984) and were approved by the South African Department of Agriculture, Forestry and Fisheries (DAFF) (Section 20 approval reference number 12/11/1/5).

### Sample collection

**Figure 1:**
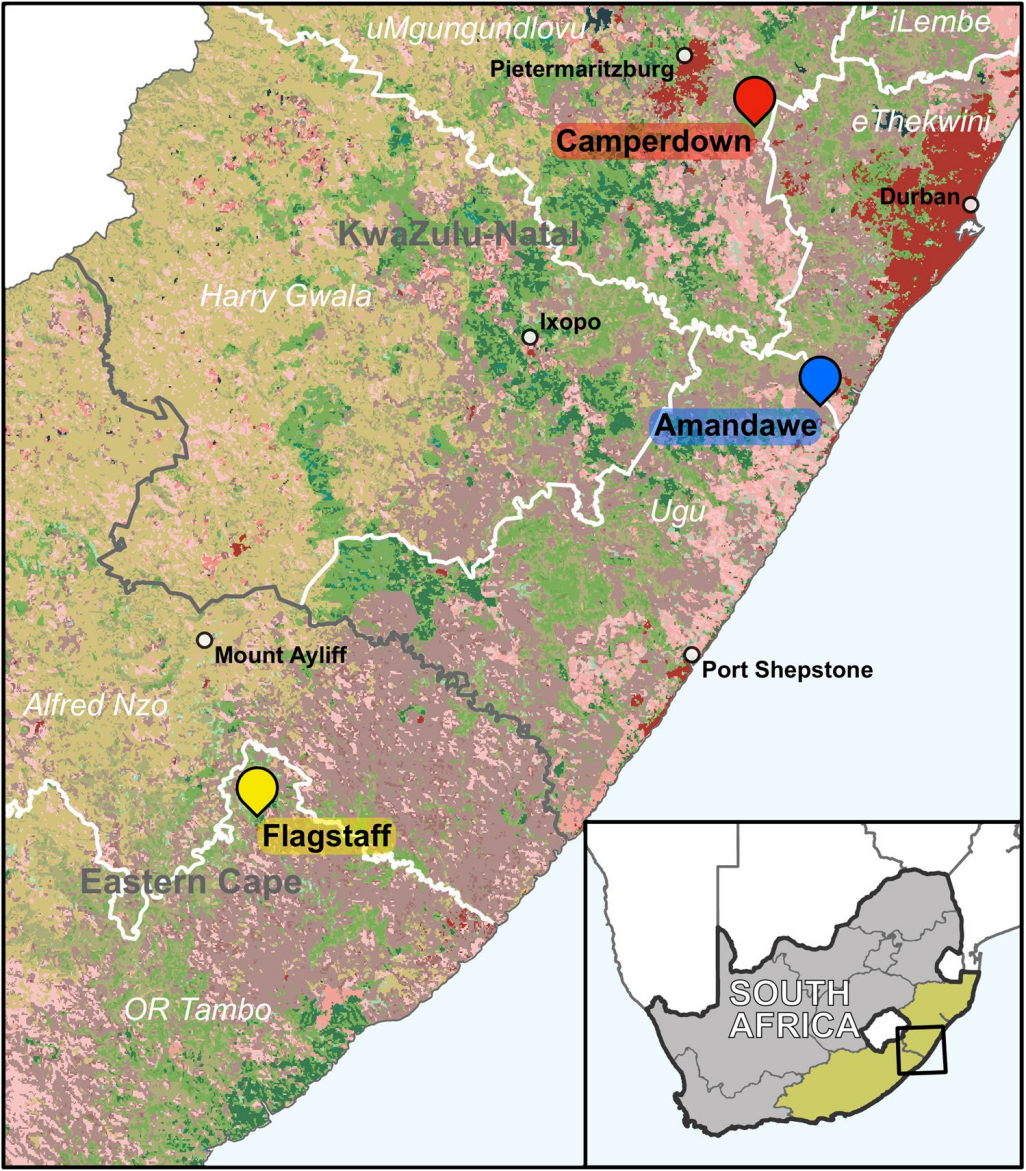
Geographic map showing the areas where the samples in the present study were collected from livestock farms in KwaZulu-Natal and Eastern Cape Provinces in South Africa. The map was generated using the software ArcGIS Pro (v3.1, Environmental Systems Research Institute (ESRI), Redlands, CA, USA).

One hundred and seventy eight samples were collected from different animals housed in livestock farms in South Africa. The livestock production farms were small-scale commercial farms located in Camperdown, Amandawe, and Scottburgh in KwaZulu-Natal (KZN) and Flagstaff in Eastern Cape (EC) Provinces as shown in Fig. 1. Samples were collected from chickens (n = 60), cows (n = 25), ducks (n = 3), goats (n = 29), horses (n = 2), sheep (n = 17), and pigs (n = 17). Additional environmental samples were obtained from water (n = 20), feedlot (n = 1), and soil (n = 4). Samples were collected from animals by rectal, oral and faecal swabbing. Samples were collected using sterile cotton swabs. After sampling, the swabs were placed in 10 ml of 0.1% (w/v) peptone water (Merck, South Africa). All samples were immediately transported to the laboratory maintaining a cold chain temperature during transport of the samples.

### Isolation and identification of *Enterococcus* species

A total of 1 ml peptone water sample was inoculated into 10 ml of brain heart infusion broth (Merck, South Africa) for enrichment and incubated for a duration of 18–24 h at 37 °C. The broth cultures were streaked onto bile aesculin azide agar (Merck, South Africa) and further incubated for a period of 18–24 h at 37 °C. Presumptive *Enterococcu*s isolates with brownish-black to black dew drop phenotype colonies were purified and isolated using trypticase soy agar (Merck, South Africa). The isolates were cultured on sheep blood agar for 18–24 h at 37 °C in the presence of 5% CO_2_. Pure colonies were suspended in trypticase soy broth to give an inoculum density equivalent to that of a 0.5 McFarland standard. An aliquot of overnight cultures of *Enterococcus* isolates in brain heart infusion broth was used for matrix-assisted laser desorption ionization–time of flight mass spectrometry (MALDI-TOF MS) analysis for species identification as previously described^25^.

### Antimicrobial susceptibility testing

Antimicrobial susceptibility testing was performed against 12 different antibiotics using Kirby-Bauer method and results were interpreted according to the standard breakpoints for inhibition zone provided by Clinical and Laboratory Standard Institute as previously described^26^.

Briefly, the bacterial isolates were grown overnight in Mueller Hinton broth (Fisher Scientific Inc., TX, USA) at 37˚C. The isolates from the overnight culture were then diluted to an optical density (OD_600_) of 0.05 (2.5 × 10^7^ colony forming unit per ml) in Mueller–Hinton broth. Approximately, a volume of 100 µl of the diluted bacteria was spread evenly on Mueller–Hinton agar plates. Antimicrobial disks were purchased from Hardy Diagnostics (Santa Maria, CA, USA) and each isolate was tested against ampicillin (10 µg, AMP 10), chloramphenicol (30 µg, C 30), azithromycin (15 µg, AZM 15), gentamicin (10 µg, GM 10), tetracycline (30 µg, TE 30), ciprofloxacin (5 µg, CIP 5), tigecycline (15 µg, TGC 15), vancomycin (30 µg, V 30), oxacillin (1 µg, OX 1), trimethoprim/sulfamethoxazole (25 µg, SXT 25), imipenem-relebactam (IMR 10/25), and levofloxacin (5 µg, LVX 5). The bacterial growth inhibition zone diameter (in mm) was measured after incubation of the bacterial isolates with the antibiotic discs at 37˚C for 12 h as previously reported^27^.

### Whole genome sequencing

Whole genome sequencing of the bacterial isolates was performed as previously reported^25,27^. Briefly, an aliquot of the bacterial culture in tryptic soy broth was diluted with 0.85% sterile saline solution to the desired inoculum density of 1 × 10^6^ CFU per ml using Thermo Scientific™ Sensititre™ Nephelometer and the chilled culture tubes were submitted for DNA extraction and sequencing. DNA extraction was performed using the Qiagen DNeasy Blood & Tissue kit (Lucigen, WI, USA) according to the manufacturer’s protocol as was previously described^20^. Sequencing libraries were prepared using the Nextera XT library preparation kit (Illumina Inc., CA, USA). Sequencing was performed on the Illumina MiSeq platform (Illumina Inc., CA, USA) using the v2 reagent kit (2 × 250 nt paired-end chemistry), which yielded 250-bp paired-end reads.

### Bioinformatics analyses

Quality control, assembly of the raw reads and virulence gene determination were performed using the ASA^3^P pipeline^28^. The raw sequence data and the assembled contigs were uploaded to the NCBI database under the accession numbers listed in Supplementary Table 1. Analysis of antibiotic resistance genes (ResFinder 2.2) and multilocus sequence type (MLST 1.6) were performed using the CGE pipeline^29^. The PlasmidFinder tool available from the Center for Genomic Epidemiology (https://cge.cbs.dtu.dk/services) was used to identify plasmid incompatibility groups. Type (Strain) Genome Server (TYGS, https://tygs.dsmz.de/, Leibniz Institute DSMZ-German Collection of Microorganisms and Cell Cultures GmbH, Braunschweig, Germany) pipeline was used to verify the species determined by MALDI-TOF^30^.

Core-genome phylogeny was determined using ParSNP^31^. The phylogenetic trees were annotated using iTOL v6.7^32,33^. Single nucleotide polymorphisms (SNPs) were determined using the output generated by ParSNP aligned in MEGA X^34^.

## Results and discussion

A total of 178 samples were collected from livestock production systems and were screened for *Enterococcus* using bile esculin azide agar selective media. Based on the phenotypic colony morphology of brown and a black halo shape, 24 isolates (13.5%) were presumptively identified as *Enterococcus* spp. The isolates were identified using MALDI-TOF analysis to be *E. faecalis* (n = 18), *E. faecium (n* = *3)* and *E. durans (n* = *3)*. The majority of *Enterococcus* strains (n = 23) were isolated from samples collected from four different animal species including goats (n = 5), chicken (n = 8), pigs (n = 4), cow (n = 5), and one *E. faecalis* strain was isolated from an environment water sample collected from a cow farm (Table 1). The majority of *Enterococcus* strains were isolated from KwaZulu-Natal Province (n = 19, 79%) and 16 isolates were obtained from fecal material while seven isolates were obtained from oral swabs.

**Table 1:** Sample types, host species and numbers of *E. faecalis*, *E. faecium* and *E. durans* isolated from livestock production systems in the present study.

Bacterial species	Host species	Sample type	Location	Number of *Enterococcus* isolates
*Enterococcus faecalis*	Pigs	Fecal	Flagstaff, EC	1
			Camperdown, KZN	2
	Goat	Fecal	Flagstaff, EC	1
			Scottburgh, KZN	2
		Oral	Amandawe, KZN	1
			Scottburgh, KZN	1
	Cow	Fecal	Scottburgh, KZN	2
		Environmental water	Scottburgh, KZN	1
	Chicken	Fecal	Scottburgh, KZN	4
		Oral	Scottburgh, KZN	2
			Amandawe, KZN	1
*Enterococcus faecium*	Cow	Fecal	Flagstaff, EC	1
	Horse	Fecal	Flagstaff, EC	1
	Chicken	Oral	Scottburgh, KZN	1
*Enterococcus durans*	Pigs	Oral	Flagstaff, EC	1
	Goat	Fecal	Scottburgh, KZN	1
	Cow	Fecal	Scottburgh, KZN	1

**Figure 2:**
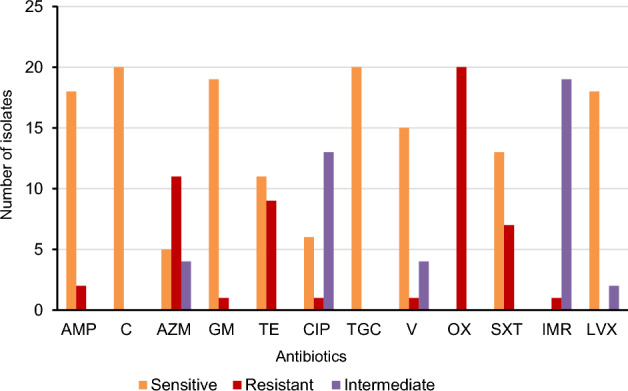
Antibiotic susceptibility patterns of *Enterococcus* spp. isolates obtained from livestock in the present study.

Antimicrobial susceptibility results showed that isolates were resistant to oxacillin (100%), tetracycline (45%, n = 9), sulfamethoxazole/trimethoprim (35%, n = 7), ampicillin (10%, n = 2), ciprofloxacin, vancomycin, and gentamicin (5%, n = 1). Isolates were intermediate resistant to ciprofloxacin (65%) and vancomycin (20%). Isolates were susceptible to chloramphenicol (100%) and levofloxacin (90% susceptible) as shown in Fig. 2. Multidrug resistance (resistance to three or more different classes of antibiotics) was observed in nine *E. faecalis* isolates and one *E. faecium* isolate. Among the tested isolates, 30% (n = 6) were resistant to two antibiotics and 50% (n = 10) isolates were resistant to more than two antibiotics, as shown in Table 2.

**Table 2:** Antibiotic resistance patterns, sequence types, and AMR genes identified in *E. fecalis* isolates in the present study.

PubMLST ID	Isolate ID	Host	MLST	AMR genes	Phenotypic antibiotic resistance pattern
2325	MEZEF110	Pig	ST4	*lsa(A)*	OX
2326	MEZEF121	Chicken	ST245	*lsa(A)*, *erm(B)*	AZM, OX
2327	MEZEF124	Chicken	ST245	*lsa(A)*, *erm(B)*	AMP, V, OX
2328	MEZEF126	Chicken	ST300	*lsa(A)*, *dfrG*, *erm(B)*, *tet(L)*, *tet(M)*	AZM, TE, OX, SXT
2329	MEZEF128	Chicken	ST93	*lsa(A)*, *aph(3′)-IIIa*, *dfrG*, *tet(L)*, *tet(M)*	AZM, TE, OX, SXT
2330	MEZEF130	Broiler chicken	ST1240	*lsa(A)*	AZM, OX, SXT
2331	MEZEF132	Broiler chicken	ST931	*lsa(A)*	OX
2332	MEZEF152	Goat	ST1241	*lsa(A)*	AMP, OX, SXT
2333	MEZEF158	Goat	ST931	*lsa(A)*	OX
2334	MEZEF159	Goat	ST1242	*lsa(A)*, *erm(B)*, *tet(L)*, *tet(M)*	AZM, TE, OX
2335	MEZEF162	Cow	ST1242	*lsa(A)*, *erm(B)*, *tet(L)*, *tet(M)*	AZM, TE, OX
2336	MEZEF164	Cow	ST1240	*lsa(A)*	AZM, OX
2337	MEZEF166	Cow	ST1240	*lsa(A)*	AZM, OX
2338	MEZEF181	Pig	ST16	*lsa(A)*, *dfrG*, *ant(6)-Ia*, *aph(3′)-IIIa*, *erm(B)*, *lnu(B)*, *sat4*, *spw*, *tet(M)*	AZM, TE, OX, SXT, GM
2339	MEZEF183	Pig	ST32	*lsa(A)*, *ant(6)-Ia*, *aph(3′)-IIIa*, *erm(B)*, *lnu(B)*, *lsa(E)*, *sat4*, *spw*, *tet(L)*, *tet(M)*	AZM, TE, OX

*Enterococcus* isolates in the present study displayed high level of resistance to tetracycline (n = 45%) (confirmed by both Kirby-Bauer method and genome sequencing analysis) and macrolides (confirmed by the detection of genes encoding macrolides resistance). The results of the present study were similar to a previous study conducted in KwaZulu-Natal Province, South Africa which reported high level of resistance to tetracycline and erythromycin (macrolide) in *Enterococcus* isolated form poultry samples^35^.

Similar results may be associated with the extensive use of antibiotics in animal production systems, similarity in the geographical region and antibiotic resistance patterns. As a result of the emergence of antibiotic resistance patterns linked to the excessive use of antibiotics in food animals, the European Union implemented a ban on the use of most of these antibiotics as animal feed additives and growth promoters^36^.

Epidemiological evidence suggests an association between the occurrence of antibiotic resistance in bacterial infections in humans and animal production systems. In many cases such as in *Enterococcus*, for example, high level of resistance to vancomycin in humans has been linked with the cross resistance from avoparcin, a vancomycin analogue used as feed additive in poultry, pig, and cattle farms^37^. It was reported that *Enterococcus* can harbor vancomycin resistance genes (*vanA*, *vanB ,vanC)* even after years of use of avoparcin as feed additive^38^.

**Figure 3:**
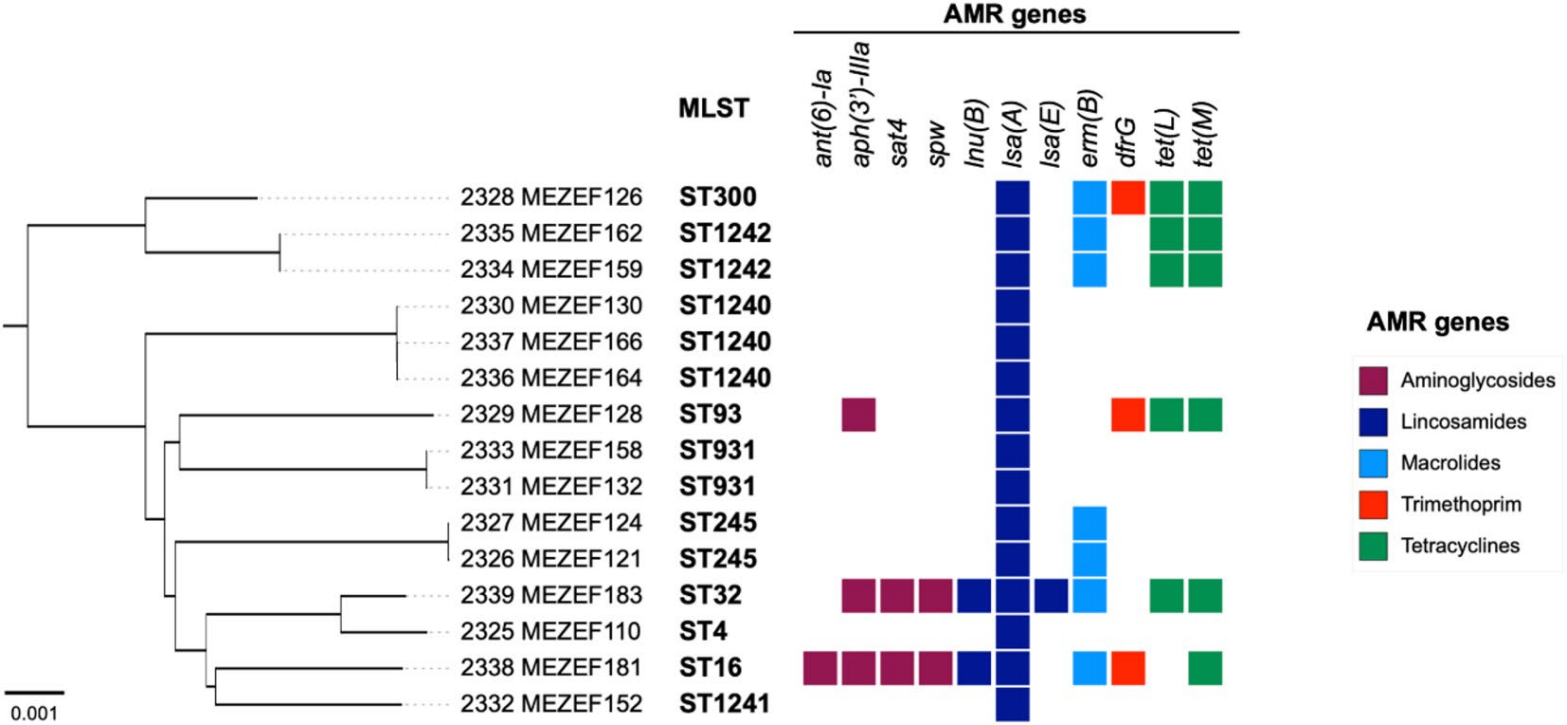
Phylogeny of *E. faecalis* isolates in the present study showing the distribution of MLST profiles and the detected antimicrobial resistance genes.

In the present study, 20 out of 24 *Enterococcus* isolates were sequenced. Three *Enterococcus faecalis* isolates and one *Enterococcus durans *isolate were only identified using MALDI-TOF analysis. Using the whole genome sequencing and TYGS, the 20 *Enterococcus* isolates were verified to be of three different *Enterococcus* species: *Enterococcus faecalis* (n = 15), *Enterococcus faecium* (n = 3) and *Enterococcus durans* (n = 2). Phylogenetic analysis was performed and phylogenetic trees of the *E. faecalis* isolates from the present study were constructed using the neighbor-joining algorithm and 1000 bootstrap replicated genomes after alignment of the genomes (Fig. 3) using Roary v3.11.2^31^.

The multilocus sequence type (ST) of each *Enterococcus faecalis* isolate was determined using the *Enterococcus faecalis* PubMLST database (https://pubmlst.org/organisms/enterococcus-faecalis). Ten STs were identified in this collection (Table 2), three of which have not previously been described (ST1240, ST1241, and ST1242). The MLST alleles and distribution of STs of each *Enterococcus faecalis* isolate are listed in Table 2.

The ST of *Enterococcus faecium* isolates (n = 3) was determined using the *Enterococcus faecium* scheme in PubMLST (https://pubmlst.org/organisms/enterococcus-faecium). Two different STs were detected, ST195 and ST2155 (Table 3). ST2155 represents a new ST type, never described before. For *Enterococcus durans*, no MLST determination could be performed due to the current lack of an MLST scheme.

**Table 3:** Antimicrobial resistance pattern, AMR genes, and ST identified in *E. faecium* and *E. durans* isolates in the present study.

PubMLST ID	Isolate ID*	Host	MLST	AMR genes	Phenotypic antibiotic resistance pattern
4561	MEZEF3	Cow	ST2155	aac(6*′*)-II, msr(C),	OX, SXT
4562	MEZEF24	Horse	ST2155	aac(6*′*)-II, msr(C)	OX
4563	MEZEF138	Broiler chicken	ST195	aac(6*′*)-II, erm(B), tet(L), tet(M)	AZM, TE, CIP, OX, SXT, IMR
Not submitted	MEZED145	Goat	Not defined	tet(L), tet(M)	TE, OX
Not submitted	MEZED165	Cow	Not defined	tet(L), tet(M)	TE, OX

*E. faecium isolates: MEZEF3, MEZEF24, MEZEF138; *E. durans* isolates: MEZED145, MEZED165.

The detection of antibiotic resistance genes in livestock-associated pathogens of zoonotic potential is an escalating public health concern. To determine the AMR genes, the contig harbouring each AMR gene was compared to sequences available in the GenBank/NCBI database. Table 2 shows the different AMR genes identified in each *E. faecalis* genome.

Antimicrobial resistance genes conferring resistance to aminoglycosides (*ant(6)-Ia*, *aph(3′)-IIIa*, , and *spw*), lincosamides (*lnu(B)*, *lsa(A)*, and *lsa(E)*), streptothricin (*sat4*), macrolides (*erm(B)*), trimethoprim (*dfrG*) and tetracyclines (*tet(L)* and *tet(M)*) were identified in *E. faecalis* using the ResFinder database. No vancomycin resistance (*van* cluster) genes were detected in the genome sequences reported in the present study.

*E. durans* strains genomes harbored *tet*(L) and *tet*(M)-like genes, while the genomes of *E. faecium* harbored macrolide, aminoglycoside and tetracycline resistance genes as shown in Table 3. Plasmid replicons *rep9a* (MEZEF128), *rep9b* (MEZEF181, MEZEF183), *rep9c* (MEZEF124), and *repUS43* (MEZEF126, MEZEF128, MEZEF159, MEZEF162, MEZEf181, MEZEF183) were detected in *E. faecalis*. Plasmid replicon types *rep2* (MEZEF138) and *rep18a* (MEZEF3, MEZEF24) were detected in *E. faecium*.

In isolates harbouring *tet(L)* and *tet(M)*, the *tet(L)* gene encodes an efflux pump^39^ whilst the *tet(M)* gene encodes a ribosomal protection protein^40^. Analysis revealed that isolate MEZEF128 harbored a contig (contig # 20) that encoded *dfrG*, *tet(L)* and *tet(M)*, isolate.

MEZEF159 harbored a contig (contig # 19) that encoded *erm(B)*, *tet(L)* and *tet(M)*, isolate MEZEF181 harbored a contig (contig # 61) that encoded *ant(6)-Ia*, *aph(3′)-IIIa*, *lnu(B)*, *sat4*, and *spw*, isolate MEZEF183 harbored a contig (contig # 64) that encoded *aph(3′)-IIIa*, *erm(B)* and *sat4*, and isolate MEZEF183 harbored a contig (contig # 72) that encoded *lnu(B)*, *lsa(E)* and *spw*. To investigate the polymorphisms in *parC* and *gyrA* associated with quinolone resistance, the amino acid sequences encoded by the *parC* and *gyrA* genes of each isolate were analysed for mutations conferring resistance to quinolones. No known mutations were identified in the *E. faecalis* genomes.

**Figure 4:**
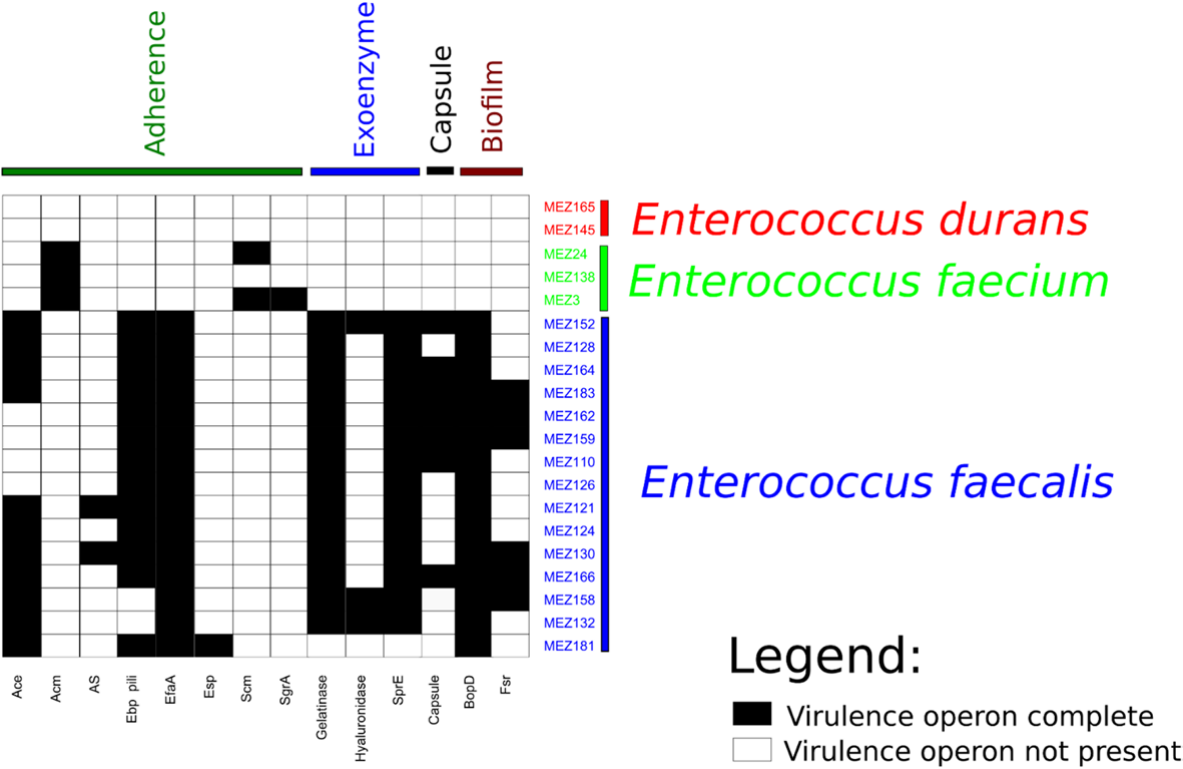
Depiction of the virulence determinants detected in *Enterococcus* spp. isolates in the present study. Only complete virulence operons are depicted.

The results of virulence determinant identification are depicted in Fig. 4. The number of virulence determinants differed by *Enterococcus* species. *E. durans* isolates did not harbor any virulence operons. *E. faecium* isolates harbored between one and three and *E. faecalis* isolates between eight to twelve virulence determinants.

The most common virulence gene types in *E. faecalis* and *E. faecium* were genes involved in adhesion. *E. faecium* harbored only virulence determinants involved in adhesion, namely *acm* (100%), *scm* (67%) and *sgrA* (34%).

*E. faecalis* genomes harbored between one to four different operons involved in adhesion, including *efaA* (100%), *ebp* pilli (87%), *ace* (73%), AS (13%) and *esp* (7%).

Three different exoenzyme genes were detected in the 15 sequenced *E. faecalis* genomes, namely gelatinase (93%), *sprE* (93%) and hyaluronidase (20%). *E. faecalis* uses these exoenzymes to degrade polymers from its host to acquire carbon and nitrogen sources for its growth.

Genes involved in capsule and biofilm production were only detected in *E. faecalis* isolates (47% and 100%, respectively). Two different genes responsible for biofilm production were detected in *E. faecalis, bopD* (15/15) and *fsr* (6/15).

The phylogenetic relationship of *E. faecalis* isolates was determined and a phylogenetic tree was constructed using the neighbor-joining algorithm after alignment of the genomes and visualization/annotation in iTOL (Fig. 3).

The determination of the closest relative of our *E. faecium* and *E. faecalis* isolates with those available in PubMLST was performed based on the STs. For *E. faecium* ST2155 and *E. faecalis* ST93, ST931, ST1240, ST1241 and ST1242, no genomes were available, thus phylogenetic relatedness could not be determined. For *E. faecium* ST195 (Supplementary Table 2) and *E. faecalis* ST4, ST16, ST32, ST245 and ST300 (Supplementary Table 3), contigs from publicly available *E. faecium* and *E. faecalis* isolates of the same ST were downloaded from the PubMLST database. Core-genome phylogeny and single nucleotide polymorphisms were determined to detect closely related isolates to those from the current study.

**Figure 5:**
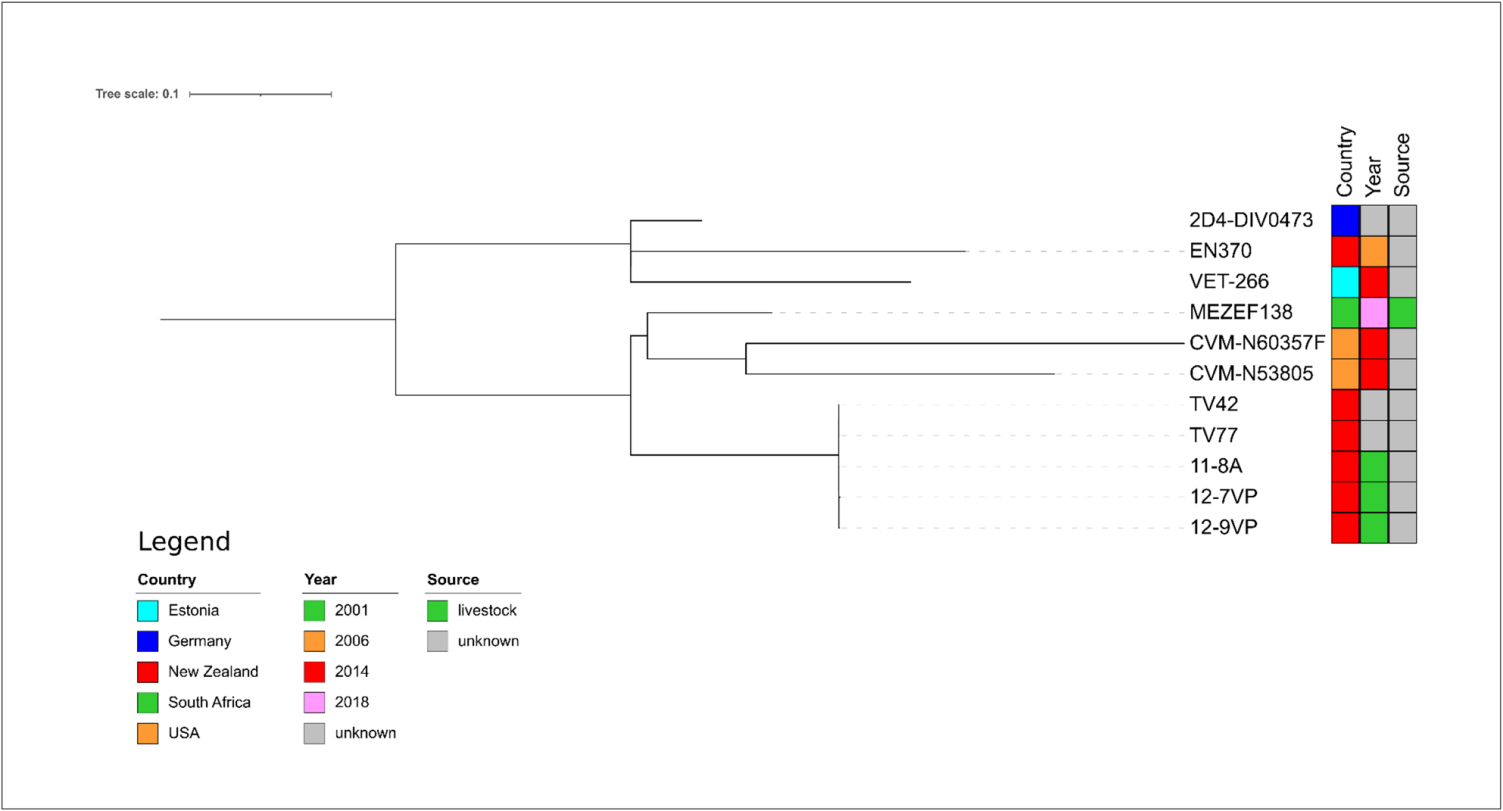
Core genome-based phylogenetic tree of MEZEF138 and all publicly available ST195 *E. faecium* genomes available in NCBI produced by the HarvestSuite package^32^, visualized using iTOL v. 6.5.8^33^, and adjusted using Inkscape 0.91 (https://inkscape.org/release/inkscape-0.91/).

For *E. faecium* ST195 isolate MEZEF138, the closest relative was the isolate TV42 (Fig. 5), an isolate detected in New Zealand from unknown source. It showed 3618 SNP differences towards MEZEF138 (Supplementary Table 4), thus indicating a very distant genetic relatedness.

**Figure 6:**
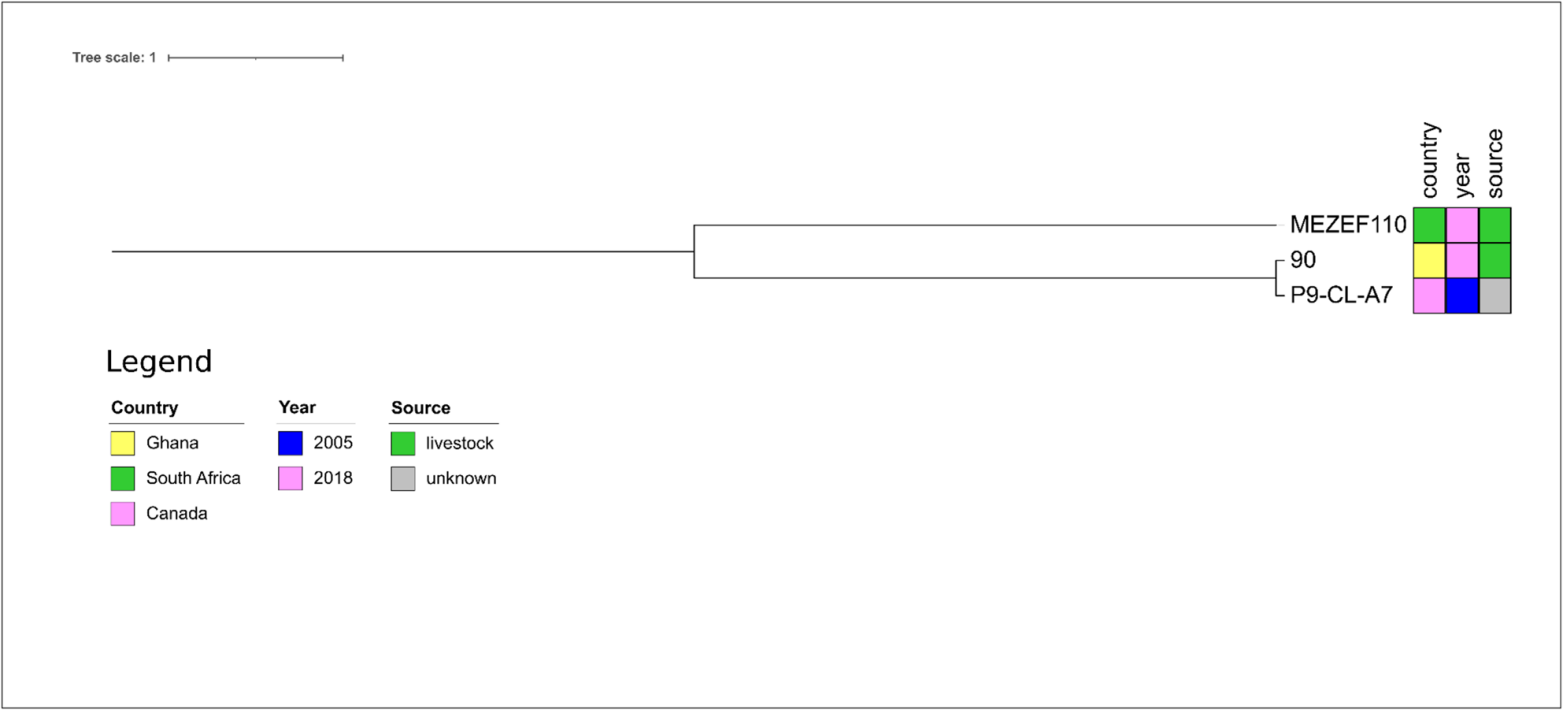
Core genome-based phylogenetic tree of MEZEF110 and all publicly available ST4 *E. faecalis* genomes available in NCBI produced by the HarvestSuite package^32^, visualized using iTOL v. 6.5.8^33^, and adjusted using Inkscape 0.91 (https://inkscape.org/release/inkscape-0.91/).

For *E. faecalis* ST4 isolate MEZEF110, the closest relative was the isolate 90 (Fig. 6) from a poultry faecal sample in Ghana with a very high number of SNPs (n = 6581, Supplementary Table 5), thus indicating a distant genetic relatedness to MEZEF110.

**Figure 7:**
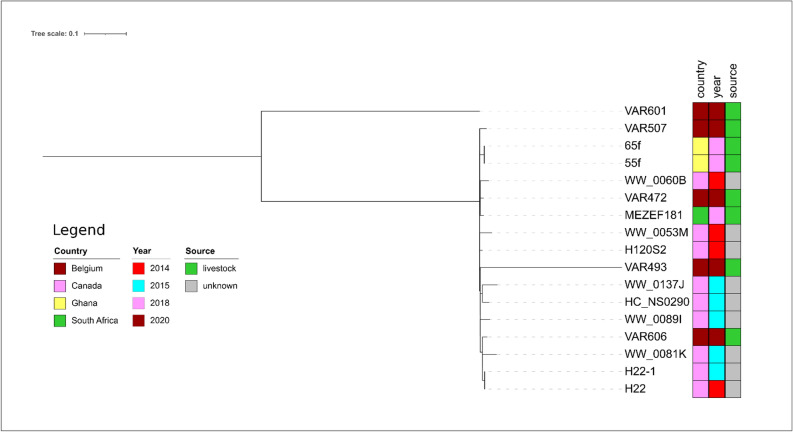
Core genome-based phylogenetic tree of MEZEF181 and all publicly available ST16 *E. faecalis* genomes available in NCBI produced by the HarvestSuite package^32^, visualized using iTOL v. 6.5.8^33^, and adjusted using Inkscape 0.91 (https://inkscape.org/release/inkscape-0.91/).

The next relatives to MEZEF181 (ST16) were the two isolates VAR472 (pig) and H120S2 (unknown source) detected in Belgium and Canada, respectively (Fig. 7). Both isolates harbored a relatively low number of SNPs as compared to MEZEF181 (n = 56, Supplementary Table 6) thus indicating a close genetic relatedness.

**Figure 8:**
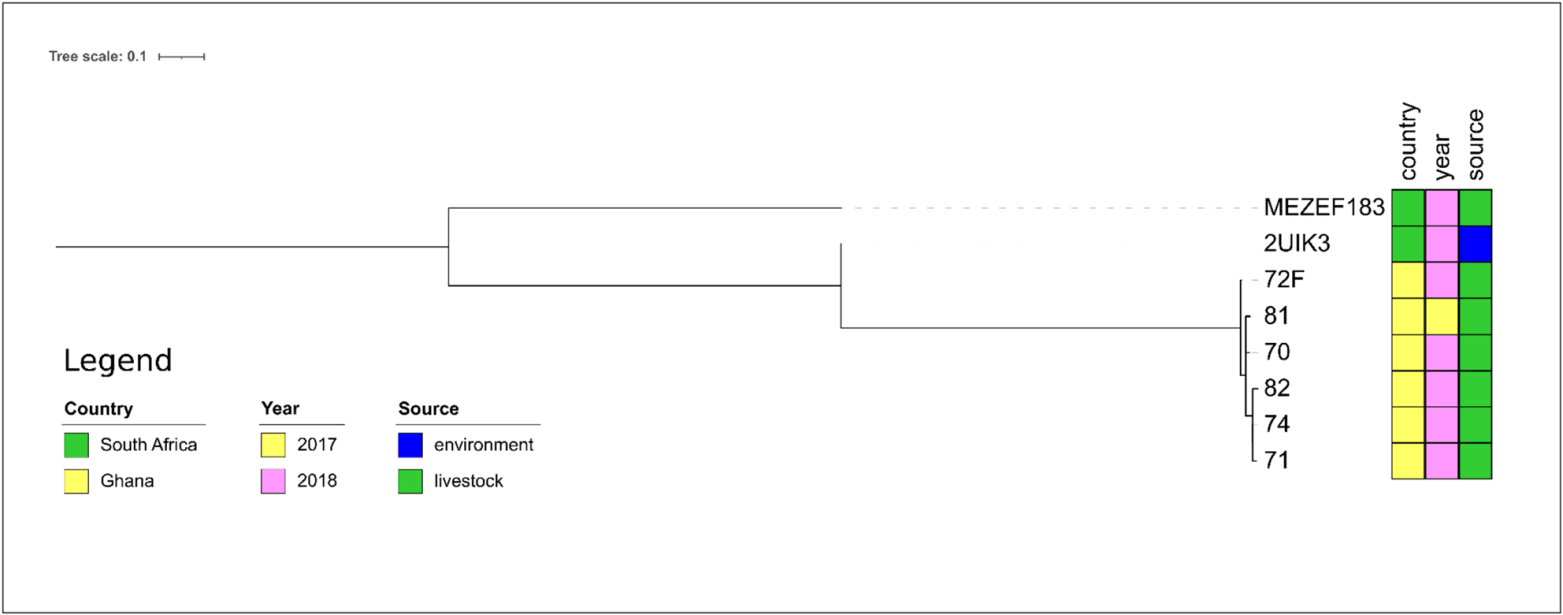
Core genome-based phylogenetic tree of MEZEF183 and all publicly available ST32 *E. fecalis* genomes available in NCBI produced by the HarvestSuite package^32^, visualized using iTOL v. 6.5.8^33^, and adjusted using Inkscape 0.91 (https://inkscape.org/release/inkscape-0.91/).

The isolate 2UIK3 from the hospital environment in South Africa was the next relative to MEZEF183 (ST32, Fig. 8), albeit the number of SNPs (n = 391, Supplementary Table 7) indicates that these two isolates are not closely related.

**Figure 9:**
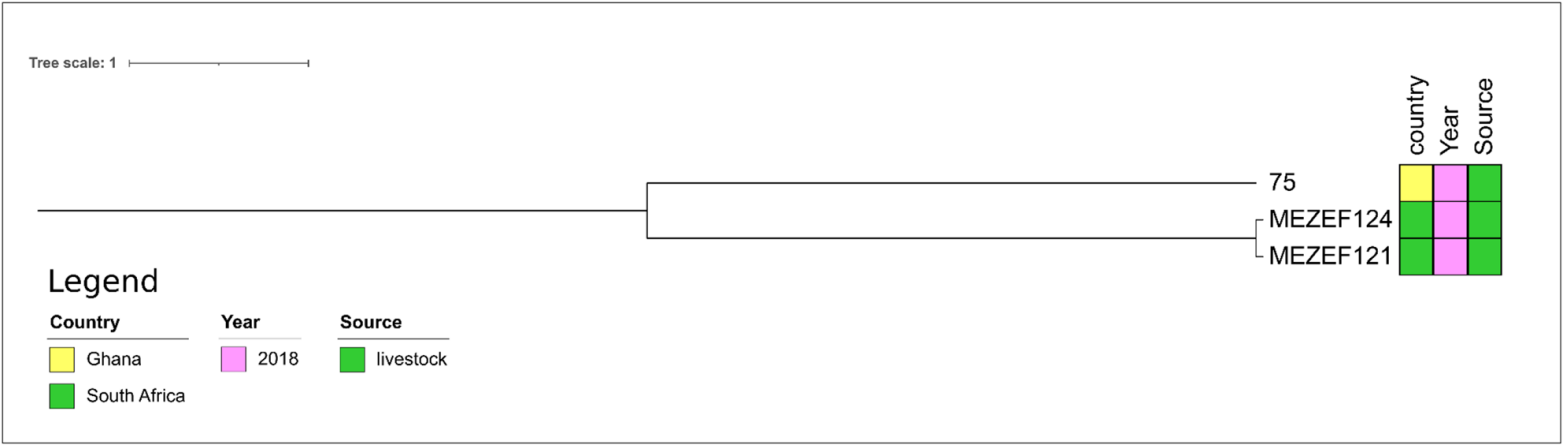
Core genome-based phylogenetic tree of MEZEF121 and MEZEF124 and all publicly available ST245 *E. faecalis* genomes available in NCBI produced by the HarvestSuite package^32^, visualized using iTOL v. 6.5.8^33^, and adjusted using Inkscape 0.91 (https://inkscape.org/release/inkscape-0.91/).

MEZEF121 and MEZEF124 (Fig. 9) were only distantly related to the only ST245 isolate designated “75” present in PubMLST (number of SNPs: n = 1450 and 1447, respectively, Supplementary Table 8). It was detected in sheep feces in Ghana.

**Figure 10:**
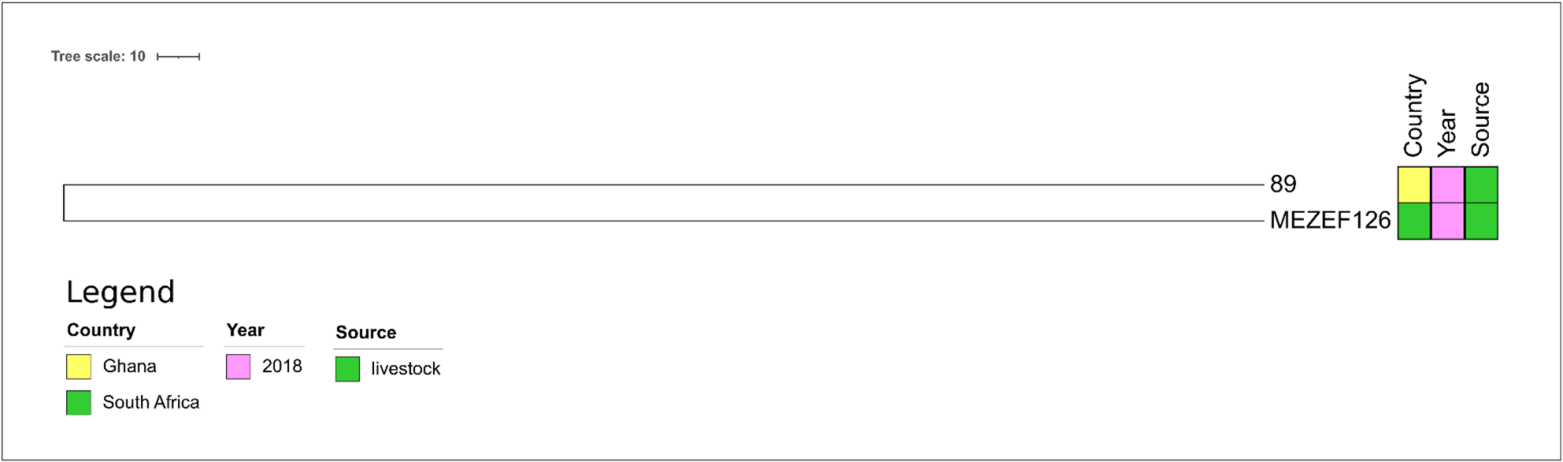
Core genome-based phylogenetic tree of MEZEF126 and all publicly available ST300 *E. faecalis* genomes available in NCBI produced by the HarvestSuite package^32^, visualized using iTOL v. 6.5.8^33^, and adjusted using Inkscape 0.91 (https://inkscape.org/release/inkscape-0.91/).

The only ST300 isolate present in PubMLST (designated 89, Fig. 10) detected in poultry feces in Ghana, was only distantly related to MEZEF126 (number of SNPs: n = 981, Supplementary Table 9).

Analysis showed that none of the *E. faecium* and *E. faecalis* from the present study belongs to a clonal complex according to the definition of clonal complex (PubMLST).

The application and implementation of whole genome sequencing in genomic surveillance efforts for antimicrobial resistance have been recently reported in clinical and animal settings^22,23,41,42^. There is a paucity of data on the role of genomic surveillance in understanding antibiotic resistance in livestock, particularly in South Africa. To the authors’ knowledge, the genomes of *Enterococcus* spp. reported in the present study are the first reported enterococcal genome sequences isolated from livestock in South Africa. Similar studies worldwide reported the detection of antimicrobial resistance in livestock-associated *Enterococcus* from diverse sources including humans, avian (chicken and turkey), swine, bovine, and ovine, their products, and the environment^43^. It was recently reported that there is an overall high prevalence of VRE (26.8%) circulating in Africa and analysis showed that a high prevalence of VRE isolated from South African region with the highest prevalence of VRE was in South Africa 74.8% (95% CI; 51–99%; I^2^ = 99.9%; *p* < 0.001) followed by, Egypt 37.2% (95% CI; − 17–92%; I^2^ = 99.7%; *p* < 0.001) and (2.8%) in Algeria and Nigeria^44^. Additionally, analysis showed a higher prevalence of VRE was isolated from environmental samples, followed by the animal source as compared to a human source^44^. In the present study, 25 samples were obtained from environmental non-animal source and analysis revealed that a single *E. fecalis *isolate (MEZEF166) of novel and previously not reported ST1240 was obtained from a water sample collected from cow’s water bucket. This finding strained the significance of the *One Health*, which is an important approach to combat antimicrobial resistance which threatens the world and causing escalated public health concerns. Vancomycin resistance determinants (*van* cluster genes) were not detected in the enterococcal genome sequences reported in the present study. The VRE phenotype of one isolate (MEZEF124) in the present study may be explained by the natural-intrinsic antibiotic resistance which are numerous in this bacterial genus^45–47^. Our results coincide with another study conducted with aquatic environmental samples in South Africa where none of the tested samples were positive for vancomycin resistance genes however the genes encoding aminoglycosides (*aph (3)-IIIa*), macrolides (*ermB*) and tetracycline resistance (*tetM* and *tetL*) were detected by PCR which coincides with results of the present study^48^. However, a previous study reported the detection of vancomycin resistance genes (*vanB* and *vanC1*/*C2/C3*) in *Enterococcus* spp. isolates using PCR in pig dung in the Eastern Cape Province in South Africa^43^. Similarly, another study also reported the detection of vancomycin resistance genes *vanC2/C3*, *vanC1* and *vanB* using PCR in fecal samples obtained from dairy cattle in South Africa^49^. This may be explained due to differences in sample source, time of sample collection, or changes in resistance trends and antimicrobial use practices. The same study also reported the detection of macrolide resistance gene *erm(B)* which coincides with the current study. Understanding the prevalence of bacteria and their antibiotic resistance patterns in food-producing animals such as dairy cattle, poultry, and pigs is crucial for gaining a comprehensive understanding of the potential health risks associated with farm-to-fork^49,50^.

To the best of the authors’ knowldege, no study has been conducted to date reporting the whole genome sequencing and sequence type of *Enterococcus* isolated from livestock in South Africa. In conclusion, the present study highlights the significance of continued genomic surveillance for antimicrobial resistance of livestock associated *Enterococcus* spp., which continue to pose a serious global threat to human health and food security, primarily a leading cause of foodborne illnesses and invasive infections in humans. The study highlights the importance of food animals as principal reservoirs of multidrug resistant pathogenic *Enterococcus* spp. The use of subtherapeutic doses of antimicrobial agents in animal feeds for prophylactic and growth-promoting purposes are among the major causes of the multiple drug resistance observed in the isolates reproted in the present study. The shedding of multiple drug-resistant, animal-associated *Enterococcus* in the environment through faecal contamination is a serious concern. *Enterococcus* spp. and important food pathogens of zoonotic potential and public health risks should be regularly surveyed and monitored in livestock production systems.

### Supplementary Information

Supplementary Table S1.

Supplementary Table S2.

Supplementary Table S3.

Supplementary Table S4.

Supplementary Table S5.

Supplementary Table S6.

Supplementary Table S7.

Supplementary Table S8.

Supplementary Table S9.

## Data Availability

This Whole Genome Shotgun project has been deposited at GenBank/NCBI under BioProject PRJNA716986, BioSample accession numbers SAMN19185853-SAMN19185872, and DDBJ/ENA/GenBank accession numbers JAHHFJ000000000-JAHHEQ000000000. The version described in this paper is version JAHHFJ010000000-JAHHEQ010000000. The sequences have been submitted to the Sequence Read Archive (SRA) under the accession numbers SRR14598116-SRR14598105. In addition, the fifteen *E. faecalis* genomes were submitted to *Enterococcus fecalis* PubMLST database (PubMLST ID: 2325-2339) and *E. faecium *genomes were submitted to *E. faecium* PubMLST database (PubMLST ID: 4561, 4562, and 4563).
