# Author Correction: Antimicrobial resistance and whole genome sequencing of novel sequence types of *Enterococcus faecalis*, *Enterococcus faecium*, and *Enterococcus durans* isolated from livestock

**DOI:** 10.1038/s41598-023-46976-2

**Published:** 2023-11-14

**Authors:** Mohamed E. El Zowalaty, Bibek Lamichhane, Linda Falgenhauer, Shakeel Mowlaboccus, Oliver T. Zishiri, Stephen Forsythe, Yosra A. Helmy

**Affiliations:** 1 Veterinary Medicine and Food Security Research Group, Medical Laboratory Sciences Program, Faculty of Health Sciences, Abu Dhabi Women’s Campus, Higher Colleges of Technology, Abu Dhabi, 41012 UAE; 2 Department of Veterinary Science, Martin-Gatton College of Agriculture, Food, and Environment, University of Kentucky, Lexington, KY 40546 USA; 3 Institute of Hygiene and Environmental Medicine, Justus Liebig University Giessen, Biomedical Research Center Seltersberg, Schubertstrasse 81, 35392 Giessen, Germany; 4 Antimicrobial Resistance and Infectious Diseases Research Laboratory, College of Science, Health, Engineering and Education, Murdoch University, Murdoch, WA Australia; 5 Department of Microbiology, PathWest Laboratory Medicine-WA, Fiona Stanley Hospital, Murdoch, WA Australia; 6 Discipline of Genetics, School of Life Sciences, College of Agriculture, Engineering and Science, University of KwaZulu-Natal, Private Bag X54001, Westville, Durban, 4000 South Africa; 7 Foodmicrobe.com Ltd., Adams Hill, Keyworth, Nottingham, NG12 5GY UK; 8 Department of Zoonoses, Faculty of Veterinary Medicine, Suez Canal University, Ismailia, 41522 Egypt

Correction to: *Scientific Reports* 10.1038/s41598-023-42838-z, published online 30 October 2023

The original version of this Article contained an error in the spelling of the author Yosra A. Helmy which was incorrectly given as Yosra A. Hemly.

The original Article has been corrected.

