# Supplementary material for: Antimicrobial resistance and whole genome sequencing of novel sequence types of Enterococcus faecalis, Enterococcus faecium, and Enterococcus durans isolated from livestock
Source: Sci Rep. 2023 Oct 30;13:18609. doi: 10.1038/s41598-023-42838-z (PMC10616195; doi:10.1038/s41598-023-42838-z)
Supplement: Supplementary file 1 — Supplementary Table S1. [file 41598_2023_42838_MOESM1_ESM.pdf]

**Supplementary Table 1: Accession numbers of the *Enterococcus* spp. in this study**

| Isolate name | Species | Biosample # | SRA data accession # | GenBank assembly accession # |
|---|---|---|---|---|
| MEZEF110 | *Enterococcus faecalis* | SAMN19185855 | SRR14598104 | GCA_020592945.1 |
| MEZEF121 | *Enterococcus faecalis* | SAMN19185856 | SRR14598103 | GCA_020592875.1 |
| MEZEF124 | *Enterococcus faecalis* | SAMN19185857 | SRR14598102 | GCA_020592835.1 |
| MEZEF126 | *Enterococcus faecalis* | SAMN19185858 | SRR14598101 | GCA_020592845.1 |
| MEZEF128 | *Enterococcus faecalis* | SAMN19185859 | SRR14598100 | GCA_020592795.1 |
| MEZEF130 | *Enterococcus faecalis* | SAMN19185860 | SRR14598099 | GCA_020592775.1 |
| MEZEF132 | *Enterococcus faecalis* | SAMN19185861 | SRR14598098 | GCA_020592755.1 |
| MEZEF152 | *Enterococcus faecalis* | SAMN19185864 | SRR14598113 | GCA_020592685.1 |
| MEZEF158 | *Enterococcus faecalis* | SAMN19185865 | SRR14598112 | GCA_020592675.1 |
| MEZEF159 | *Enterococcus faecalis* | SAMN19185866 | SRR14598111 | GCA_020592635.1 |
| MEZEF162 | *Enterococcus faecalis* | SAMN19185867 | SRR14598110 | GCA_020592555.1 |
| MEZEF164 | *Enterococcus faecalis* | SAMN19185868 | SRR14598109 | GCA_020592565.1 |
| MEZEF166 | *Enterococcus faecalis* | SAMN19185870 | SRR14598107 | GCA_020592505.1 |
| MEZEF181 | *Enterococcus faecalis* | SAMN19185871 | SRR14598106 | GCA_020592455.1 |
| MEZEF183 | *Enterococcus faecalis* | SAMN19185872 | SRR14598105 | GCA_020592415.1 |
| MEZEF3 | *Enterococcus faecium* | SAMN19185853 | SRR14598116 | GCA_020593005.1 |
| MEZEF24 | *Enterococcus faecium* | SAMN19185854 | SRR14598115 | GCA_020592895.1 |
| MEZEF138 | *Enterococcus faecium* | SAMN19185862 | SRR14598097 | GCA_020592725.1 |
| MEZED145 | *Enterococcus durans* | SAMN19185863 | SRR14598114 | GCA_020592715.1 |
| MEZED165 | *Enterococcus durans* | SAMN19185869 | SRR14598108 | GCA_020592465.1 |
