# Supplementary material for: Antimicrobial resistance and whole genome sequencing of novel sequence types of Enterococcus faecalis, Enterococcus faecium, and Enterococcus durans isolated from livestock
Source: Sci Rep. 2023 Oct 30;13:18609. doi: 10.1038/s41598-023-42838-z (PMC10616195; doi:10.1038/s41598-023-42838-z)
Supplement: Supplementary file 2 — Supplementary Table S2. [file 41598_2023_42838_MOESM2_ESM.pdf]

Supplementary Table 2: Enterococcus faecium ST195 genomes downloaded from the pubMLST database

id	isolate	country	continent	year	source_category	source	detailed_source	ST (MLST)
5885	2D4_DIV0473	Germany	Europe	unknown	unknown	unknown	unknown	195
5993	CVM N53805	USA	North America	2014	unknown	unknown	unknown	195
6043	CVM N60357F	USA	North America	2014	unknown	unknown	unknown	195
6388	EN370	New Zealand	Oceania	2006	unknown	unknown	unknown	195
6444	11-8A	New Zealand	Oceania	2001	unknown	unknown	unknown	195
6448	12-7VP	New Zealand	Oceania	2001	unknown	unknown	unknown	195
6450	12-9VP	New Zealand	Oceania	2001	unknown	unknown	unknown	195
6593	TV42	New Zealand	Oceania	unknown	unknown	unknown	unknown	195
6610	TV77	New Zealand	Oceania	unknown	unknown	unknown	unknown	195
7094	VET-266	Estonia	Europe	2014	unknown	unknown	unknown	195
