# Supplementary material for: Antimicrobial resistance and whole genome sequencing of novel sequence types of Enterococcus faecalis, Enterococcus faecium, and Enterococcus durans isolated from livestock
Source: Sci Rep. 2023 Oct 30;13:18609. doi: 10.1038/s41598-023-42838-z (PMC10616195; doi:10.1038/s41598-023-42838-z)
Supplement: Supplementary file 3 — Supplementary Table S3. [file 41598_2023_42838_MOESM3_ESM.pdf]

Supplementary Table 2: ***Enterococcus faecalis*** **ST4, ST16. ST32, ST245 and ST300 genomes downloaded from the pubMLST database**

id	isolate	country	continent	region	year	source_category	source	detailed_source	ST (MLST)
2539	90	Ghana	Africa		2018	other animals	faeces	Poultry	4
2684	P9 CL A7	Canada	North America		2005	unknown			4
2176	VAR472	Belgium	Europe		2020	husbandry	animal	animal_pig	16
2185	VAR493	Belgium	Europe		2020	husbandry	animal	animal_bird	16
2193	VAR507	Belgium	Europe		2020	husbandry	animal	animal_pig	16
2241	VAR601	Belgium	Europe		2020	husbandry	animal	animal_pig	16
2243	VAR606	Belgium	Europe		2020	husbandry	animal	animal_bovine	16
2531	55f	Ghana	Africa		2018	other animals	faeces	Goat	16
2532	65f	Ghana	Africa		2018	other animals	faeces	Goat	16
2717	H22	Canada	North America		2014	unknown			16
2731	H120S2	Canada	North America		2014	unknown			16
2753	WW_0060B	Canada	North America		2014	unknown			16
2754	WW_0053M	Canada	North America		2014	unknown			16
2758	WW_0137J	Canada	North America		2015	unknown			16
2759	WW_0089I	Canada	North America		2015	unknown			16
2761	WW_0081K	Canada	North America		2015	unknown			16
2786	HC_NS0290	Canada	North America		2015	unknown			16
2801	H22-1	Canada	North America		2015	unknown			16
2508	2UIK3	South Africa	Africa	Ethekwini district	2017	environment	other	Other	32
2534	70	Ghana	Africa		2018	other animals	faeces	Goat	32
2535	71	Ghana	Africa		2018	other animals	faeces	Goat	32
2540	81	Ghana	Africa		2018	other animals	faeces	Sheep	32
2541	72F	Ghana	Africa		2018	other animals	faeces	Sheep	32
2542	74	Ghana	Africa		2018	other animals	faeces	Sheep	32
2544	82	Ghana	Africa		2018	other animals	faeces	Sheep	32
2543	75	Ghana	Africa		2018	other animals	faeces	Sheep	245
2545	89	Ghana	Africa		2018	other animals	faeces	Poultry	300
