# Supplementary material for: Antimicrobial resistance and whole genome sequencing of novel sequence types of Enterococcus faecalis, Enterococcus faecium, and Enterococcus durans isolated from livestock
Source: Sci Rep. 2023 Oct 30;13:18609. doi: 10.1038/s41598-023-42838-z (PMC10616195; doi:10.1038/s41598-023-42838-z)
Supplement: Supplementary file 4 — Supplementary Table S4. [file 41598_2023_42838_MOESM4_ESM.pdf]

Supplementary Table 4: Single nucleotide polymorphism matrix of *Enterococcus faecium* ST195 isolates.

	MEZEF138	6593_TV42	6610_TV77	6444_11_8A	6450_12_9VP	6448_12_7VP	5993_CVM_N53805	6043_CVM_N60357F	5885_2D4_DIV0473	7094_VET_266	6388_EN370
MEZEF138		3618	3619	3619	3620	3626	5100	5980	6200	7492	7995
6593_TV42	3618		1	7	10	20	6083	6763	6525	7370	8009
6610_TV77	3619	1		8	11	21	6084	6764	6526	7371	8010
6444_11_8A	3619	7	8		5	15	6084	6764	6526	7371	8010
6450_12_9VP	3620	10	11	5		16	6085	6765	6527	7374	8011
6448_12_7VP	3626	20	21	15	16		6095	6775	6537	7384	8017
5993_CVM_N53805	5100	6083	6084	6084	6085	6095		6626	8057	9882	10137
6043_CVM_N60357F	5980	6763	6764	6764	6765	6775	6626		7549	9014	8563
5885_2D4_DIV0473	6200	6525	6526	6526	6527	6537	8057	7549		3638	4050
7094_VET_266	7492	7370	7371	7371	7374	7384	9882	9014	3638		5639
6388_EN370	7995	8009	8010	8010	8011	8017	10137	8563	4050	5639	

Table. Estimates of Evolutionary Divergence between Sequences

The number of base differences per sequence from between sequences are shown. This analysis involved 11 nucleotide sequences. All positions containing gaps and missing data were eliminated (complete deletion option). There were a total of 17007 positions in the final dataset. Evolutionary analyses were conducted in MEGA X [1]

1. Kumar S., Stecher G., Li M., Knyaz C., and Tamura K. (2018). MEGA X: Molecular Evolutionary Genetics Analysis across computing platforms. Molecular Biology and Evolution 35:1547-1549.

Disclaimer: Although utmost care has been taken to ensure the correctness of the caption, the caption text is provided "as is" without any warranty of any kind. Authors advise the user to carefully check the caption prior to its use for any purpose and report any errors or problems to the authors immediately (www.megasoftware.net). In no event shall the authors and their employers be liable for any damages, including but not limited to special, consequential, or other damages. Authors specifically disclaim all other warranties expressed or implied, including but not limited to the determination of suitability of this caption text for a specific purpose, use, or application.
