# Supplementary material for: Antimicrobial resistance and whole genome sequencing of novel sequence types of Enterococcus faecalis, Enterococcus faecium, and Enterococcus durans isolated from livestock
Source: Sci Rep. 2023 Oct 30;13:18609. doi: 10.1038/s41598-023-42838-z (PMC10616195; doi:10.1038/s41598-023-42838-z)
Supplement: Supplementary file 5 — Supplementary Table S5. [file 41598_2023_42838_MOESM5_ESM.pdf]

Supplementary Table 5: Single nucleotide polymorphism matrix of *Enterococcus faecalis* ST4 isolates.

	MEZEF110	2684_P9_CL_A7	2539_90
MEZEF110		6581	6693
2539_90	6581		553
2684_P9_CL_A7	6693	553	

Table. Estimates of Evolutionary Divergence between Sequences

The number of base differences per sequence from between sequences are shown. This analysis involved 3 nucleotide sequences. All positions containing gaps and missing data were eliminated (complete deletion option). There were a total of 6912 positions in the final dataset. Evolutionary analyses were conducted in MEGA X [1]

1. Kumar S., Stecher G., Li M., Knyaz C., and Tamura K. (2018). MEGA X: Molecular Evolutionary Genetics Analysis across computing platforms. Molecular Biology and Evolution 35:1547-1549.

Disclaimer: Although utmost care has been taken to ensure the correctness of the caption, the caption text is provided "as is" without any warranty of any kind. Authors advise the user to carefully check the caption prior to its use for any purpose and report any errors or problems to the authors immediately (www.megasoftware.net). In no event shall the authors and their employers be liable for any damages, including but not limited to special, consequential, or other damages. Authors specifically disclaim all other warranties expressed or implied, including but not limited to the determination of suitability of this caption text for a specific purpose, use, or application.
