# Supplementary material for: Antimicrobial resistance and whole genome sequencing of novel sequence types of Enterococcus faecalis, Enterococcus faecium, and Enterococcus durans isolated from livestock
Source: Sci Rep. 2023 Oct 30;13:18609. doi: 10.1038/s41598-023-42838-z (PMC10616195; doi:10.1038/s41598-023-42838-z)
Supplement: Supplementary file 6 — Supplementary Table S6. [file 41598_2023_42838_MOESM6_ESM.pdf]

Supplementary Table 6: Single nucleotide polymorphism matrix of *Enterococcus faecalis* ST16 isolates.

	2338_MEZEF181	2176_VAR472	2731_H120S2	2531_55f	2532_65f	2717_H22	2801_H22_1	2193_VAR507	2243_VAR606	2753_WW_0060B	2786_HC_NS0290	2759_WW_0089I	2754_WW_0053M	2761_WW_0081K	2758_WW_0137J	2185_VAR493	2241_VAR601
2338_MEZEF181		56	56	70	70	76	78	88	96	99	111	118	134	174	180	1018	2136
2176_VAR472	56		56	70	70	74	76	88	96	99	111	118	134	174	180	1019	2136
2731_H120S2	56	56		62	62	66	68	80	86	97	101	108	124	164	170	1009	2126
2531_55f	70	70	62		0	82	84	94	102	111	117	124	140	180	186	1023	2142
2532_65f	70	70	62	0		82	84	94	102	111	117	124	140	180	186	1023	2142
2717_H22	76	74	66	82	82		2	100	58	117	111	118	144	136	180	1019	2144
2801_H22_1	78	76	68	84	84	2		102	60	119	113	120	146	138	182	1021	2146
2193_VAR507	88	88	80	94	94	100	102		120	129	135	142	158	198	204	1043	2160
2243_VAR606	96	96	86	102	102	58	60	120		137	131	138	164	156	200	1039	2162
2753_WW_0060B	99	99	97	111	111	117	119	129	137		152	159	174	215	221	1060	2177
2786_HC_NS0290	111	111	101	117	117	111	113	135	131	152		153	179	209	179	1054	2179
2759_WW_0089I	118	118	108	124	124	118	120	142	138	159	153		185	216	222	1060	2186
2754_WW_0053M	134	134	124	140	140	144	146	158	164	174	179	185		242	248	1087	2203
2761_WW_0081K	174	174	164	180	180	136	138	198	156	215	209	216	242		274	1115	2241
2758_WW_0137J	180	180	170	186	186	180	182	204	200	221	179	222	248	274		1123	2248
2185_VAR493	1018	1019	1009	1023	1023	1019	1021	1043	1039	1060	1054	1060	1087	1115	1123		2871
2241_VAR601	2136	2136	2126	2142	2142	2144	2146	2160	2162	2177	2179	2186	2203	2241	2248	2871	
