# Supplementary material for: Antimicrobial resistance and whole genome sequencing of novel sequence types of Enterococcus faecalis, Enterococcus faecium, and Enterococcus durans isolated from livestock
Source: Sci Rep. 2023 Oct 30;13:18609. doi: 10.1038/s41598-023-42838-z (PMC10616195; doi:10.1038/s41598-023-42838-z)
Supplement: Supplementary file 7 — Supplementary Table S7. [file 41598_2023_42838_MOESM7_ESM.pdf]

Supplementary Table 7: Single nucleotide polymorphism matrix of *Enterococcus faecalis* ST32 isolates.

	MEZEF183	2508_2UIK3	2534_70	2540_81	2541_72F	2542_74	2535_71	2544_82
MEZEF183		391	453	454	456	457	461	463
2508_2UIK3	391		294	295	287	298	302	294
2534_70	453	294		9	11	12	16	18
2540_81	454	295	9		12	13	11	19
2541_72F	456	287	11	12		15	19	11
2542_74	457	298	12	13	15		4	6
2535_71	461	302	16	11	19	4		10
2544_82	463	294	18	19	11	6	10	

Table. Estimates of Evolutionary Divergence between Sequences

The number of base differences per sequence from between sequences are shown. This analysis involved 8 nucleotide sequences. All positions containing gaps and missing data were eliminated (complete deletion option). There were a total of 585 positions in the final dataset. Evolutionary analyses were conducted in MEGA X [1]

1. Kumar S., Stecher G., Li M., Knyaz C., and Tamura K. (2018). MEGA X: Molecular Evolutionary Genetics Analysis across computing platforms. Molecular Biology and Evolution 35:1547-1549.

Disclaimer: Although utmost care has been taken to ensure the correctness of the caption, the caption text is provided "as is" without any warranty of any kind. Authors advise the user to carefully check the caption prior to its use for any purpose and report any errors or problems to the authors immediately (www.megasoftware.net). In no event shall the authors and their employers be liable for any damages, including but not limited to special, consequential, or other damages. Authors specifically disclaim all other warranties expressed or implied, including but not limited to the determination of suitability of this caption text for a specific purpose, use, or application.
